# Quantitative flow ratio to predict long-term coronary artery bypass graft patency in patients with left main coronary artery disease

**DOI:** 10.1007/s10554-022-02699-5

**Published:** 2022-08-28

**Authors:** Cameron Dowling, Adam J. Nelson, Ren Yik Lim, Jun Michael Zhang, Kevin Cheng, Julian A. Smith, Sujith Seneviratne, Yuvaraj Malaiapan, Sarah Zaman, Dennis T. L. Wong

**Affiliations:** 1 MonashHeart, Monash Health and Monash Cardiovascular Research Centre, Monash University, Melbourne, Australia; 2 Division of Cardiovascular Medicine, Stanford University School of Medicine, Stanford, CA USA; 3 Duke Clinical Research Institute, Duke University, Durham, NC USA; 4 Department of Cardiothoracic Surgery, Monash Health and Department of Surgery (School of Clinical Sciences at Monash Health), Monash University, Melbourne, Australia; 5 Department of Cardiology, Westmead Hospital and Westmead Applied Research Centre, University of Sydney, Sydney, Australia; 6 School of Clinical Sciences at Monash Health, Monash University, Melbourne, Australia; 7 MonashHeart, 246 Clayton Rd, Clayton, VIC 3168 Australia

**Keywords:** Coronary artery bypass grafting, Graft occlusion, Computed tomography coronary angiography, Quantitative flow reserve

## Abstract

**Purpose:**

Fractional flow reserve (FFR) has been demonstrated in some studies to predict long-term coronary artery bypass graft (CABG) patency. Quantitative flow ratio (QFR) is an emerging technology which may predict FFR. In this study, we hypothesised that QFR would predict long-term CABG patency and that QFR would offer superior diagnostic performance to quantitative coronary angiography (QCA) and intravascular ultrasound (IVUS).

**Methods:**

A prospective study was performed on patients with left main coronary artery disease who were undergoing CABG. QFR, QCA and IVUS assessment was performed. Follow-up computed tomography coronary angiography and invasive coronary angiography was undertaken to assess graft patency.

**Results:**

A total of 22 patients, comprising of 65 vessels were included in the analysis. At a median follow-up of 3.6 years post CABG (interquartile range, 2.3 to 4.8 years), 12 grafts (18.4%) were occluded. QFR was not statistically significantly higher in occluded grafts (0.81 ± 0.19 vs. 0.69 ± 0.21; P = 0.08). QFR demonstrated a discriminatory power to predict graft occlusion (area under the receiver operating characteristic curve, 0.70; 95% confidence interval [CI], 0.52 to 0.88; P = 0.03). At long-term follow-up, the risk of graft occlusion was higher in vessels with a QFR > 0.80 (58.6% vs. 17.0%; hazard ratio, 3.89; 95% CI, 1.05 to 14.42; P = 0.03 by log-rank test). QCA (minimum lumen diameter, lesion length, diameter stenosis) and IVUS (minimum lumen area, minimum lumen diameter, diameter stenosis) parameters were not predictive of long-term graft patency.

**Conclusions:**

QFR may predict long-term graft patency in patients undergoing CABG.

## Introduction

Fractional flow reserve (FFR) has been demonstrated to predict both short and long-term coronary artery bypass graft (CABG) patency, as well as a number of important clinical endpoints, including death and myocardial infarction [1, 2]. However, it would be desirable to assess the physiological significance of coronary stenoses in patients being considered for CABG without the need for either wire-based tools or administration of vasoactive medications, especially for patients with left main coronary artery (LMCA) disease, where procedural risks may be elevated.

Quantitative flow ratio (QFR) is an established method for predicting the functional significance of coronary lesions [3], and this technology may assist in guiding revascularisation strategies and provide prognostication for patients with LMCA disease [4]. QFR is derived using complex mathematical methods built upon the principles of computational fluid dynamics and is calculated using a modelled hyperaemic flow velocity, derived from thrombolysis in myocardial infarction (TIMI) frame count analysis, without pharmacologically-induced hyperaemia [5].

In this study, we hypothesised that QFR would predict long-term graft patency for patients with LMCA disease. Furthermore, we hypothesised that QFR would offer superior diagnostic performance to angiographic and intravascular ultrasound (IVUS) parameters for predicting long-term graft patency.

## Methods

A prospective single-centre study was performed on patients with angiographically moderate (50–70%) LMCA disease who had undergone IVUS assessment as part of their diagnostic evaluation for CABG. Patients with downstream disease were not excluded from the study. The primary endpoint was long-term graft patency as assessed by computed tomography coronary angiography (CTCA) with supplementation from invasive coronary angiography.

Patients provided informed consent and the study protocol was approved by a local human research ethics committee. Baseline patient characteristics was ascertained from local electronic records and used to calculate EuroSCORE II [6].

### Intravascular ultrasound

IVUS assessment of the LMCA was performed using an OptiCross (Boston Scientific, Marlborough, MA) IVUS catheter with the assistance of an automatic pullback sled. Intracoronary glyceryl trinitrate was administered, and care taken to ensure guiding catheter disengagement during recordings. Minimum lumen area (MLA), minimum lumen diameter (MLD) and stenosis percentage were measured. IVUS assessment of downstream vessels was not undertaken.

### Quantitate flow ratio analysis

QFR analysis was undertaken on all grafted vessels, including right-sided vessels, using QAngio XA 3D v3.1.1 (Medis Medical Imaging System, Leiden, The Netherlands) by an independent operator, blinded to clinical endpoint information. Analysis was performed on two angiographic acquisitions that were separated by ≥ 25°, ensuring that the angiographic projections had minimal foreshortening of the stenosis, and only minimal overlap of the main vessel and the side branches. Vessel QFR was recorded. QFR was not performed on vessels that were not grafted. Two-dimensional quantitative coronary angiography (QCA) was performed, and percentage diameter stenosis, lesion length and minimum lumen diameter recorded.

### Determination of graft patency

Computed tomography coronary angiography (CTCA) was undertaken 5 years following CABG using an Aquilion ONE ViSION 320-slice scanner (Canon Medical Systems Corporation, Otawara, Japan). Vessel analysis was performed by experienced readers in CTCA who were blinded to QFR values. CTCA and invasive coronary angiography which were undertaken for clinical purposes were also reviewed to provide additional information on long-term graft patency. Grafts were defined as occluded if they were either completely occluded or atretic.

### Statistical analysis

Statistical analysis was performed using SPSS version 28.0 (IBM Corporation, Armonk, NY). Continuous variables are presented as mean ± standard deviation and categorical variables as frequencies (percentage). The means of groups were compared with a two-tailed Student’s t-test with a P value < 0.05 considered statistically significant. Contingency analysis on two groups was performed using a Fisher’s exact test, and on more than two groups with a chi-squared test. Potential predictors for clinical outcomes were assessed using univariate binary logistic regression analysis. Time-to-event analysis was performed with the use of Kaplan–Meier estimates and Cox regression and were compared with the use of the log-rank test. Correlation was assessed using a Pearson’s correlation coefficient.

## Results

### Patient characteristics

Between 2007 and 2014 a total of 22 patients were included in the study. Baseline patient characteristics are presented in Table 1. Patients were at low risk for surgery (EuroSCORE II 1.2 ± 0.3%). The majority of patients were male (77.3%) and there was a high prevalence of atherosclerotic cardiovascular disease risk factors.

**Table 1:** Baseline patient characteristics

Characteristic	N = 22
Age (years), mean ± SD	62.1 ± 9.2
Male, N (%)	17 (77.3)
Body surface area (m^2^), mean ± SD	1.95 ± 0.18
Clinical Presentation, N (%)	
Stable angina	9 (41)
Unstable angina	7 (32)
NSTEMI	4 (18)
STEMI	2 (9)
EuroSCORE II (%), mean ± SD	1.2 ± 0.3
Atherosclerotic Cardiovascular Risk Factors, N (%)	
Hypertension	11 (50.0)
Dyslipidaemia	14 (63.6)
Diabetes mellitus	8 (36.4)
Cigarette smoking	11 (50.0)
Family history of ischaemic heart disease	6 (27.3)
Left ventricular ejection fraction (%), mean ± SD	59.2 ± 5.2

*NSTEMI* Non-ST segment myocardial infarction, and *STEMI* ST-elevation myocardial infarction

### LMCA assessment

LMCA lesion distribution was ostial (9.1%), mid-body (4.5%), distal (81.8%) and diffuse (4.5%). Mean angiographic lesion severity was 55.7 ± 8.2%. On IVUS assessment, the mean MLA was 5.49 ± 1.89 mm^2^, diameter stenosis 2.26 ± 0.45 mm and percentage stenosis 64.7 ± 12.7%. An MLA < 6 mm^2^ was present in 63.6% of patients.

### Coronary artery bypass grafting procedures

All CABG procedures were performed on-pump. An average of 3.0 ± 1.0 grafts were placed and left internal mammary artery grafting of the left anterior descending artery was performed in all cases. Other coronary artery bypass conduits used included the right internal mammary artery in 14%, radial artery in 86% and saphenous vein in 55% of patients.

### QFR analysis

A total of 72 vessel were considered for QFR analysis, but 7 vessels were excluded as there were inadequate orthogonal views, leaving a total of 65 vessels for study inclusion. QFR analysis was performed at a median of 9.0 years following initial invasive coronary angiography (interquartile range, 7.6 to 9.9 years). Vessels assessed included the left anterior descending (n = 22), diagonal (n = 9), ramus intermedius (n = 6), obtuse marginal (n = 16) and posterior descending (n = 12) arteries.

The mean QFR was 0.72 ± 0.21 and 36 vessels (55.4%) had a functionally significant QFR ≤ 0.80.

### Graft patency

All patients underwent follow-up CTCA assessment, and this information was supplemented by invasive coronary angiography in 8 cases. Median follow-up was 3.6 years (interquartile range, 2.3 to 4.8 years). A total of 12 grafts (18.5%) were occluded. There was no significant difference in the incidence of graft patency amongst internal mammary artery (83.3%), radial artery (66.7%) and saphenous venous (100%) conduits (P = 0.41). Grafts were more likely to be patent when placed on the left anterior descending artery or its sub-branches (90.3%), when compared to the circumflex (78.2%) or right coronary artery (63.6%) and their associated sub-branches (P = 0.04).

**Fig. 1:**
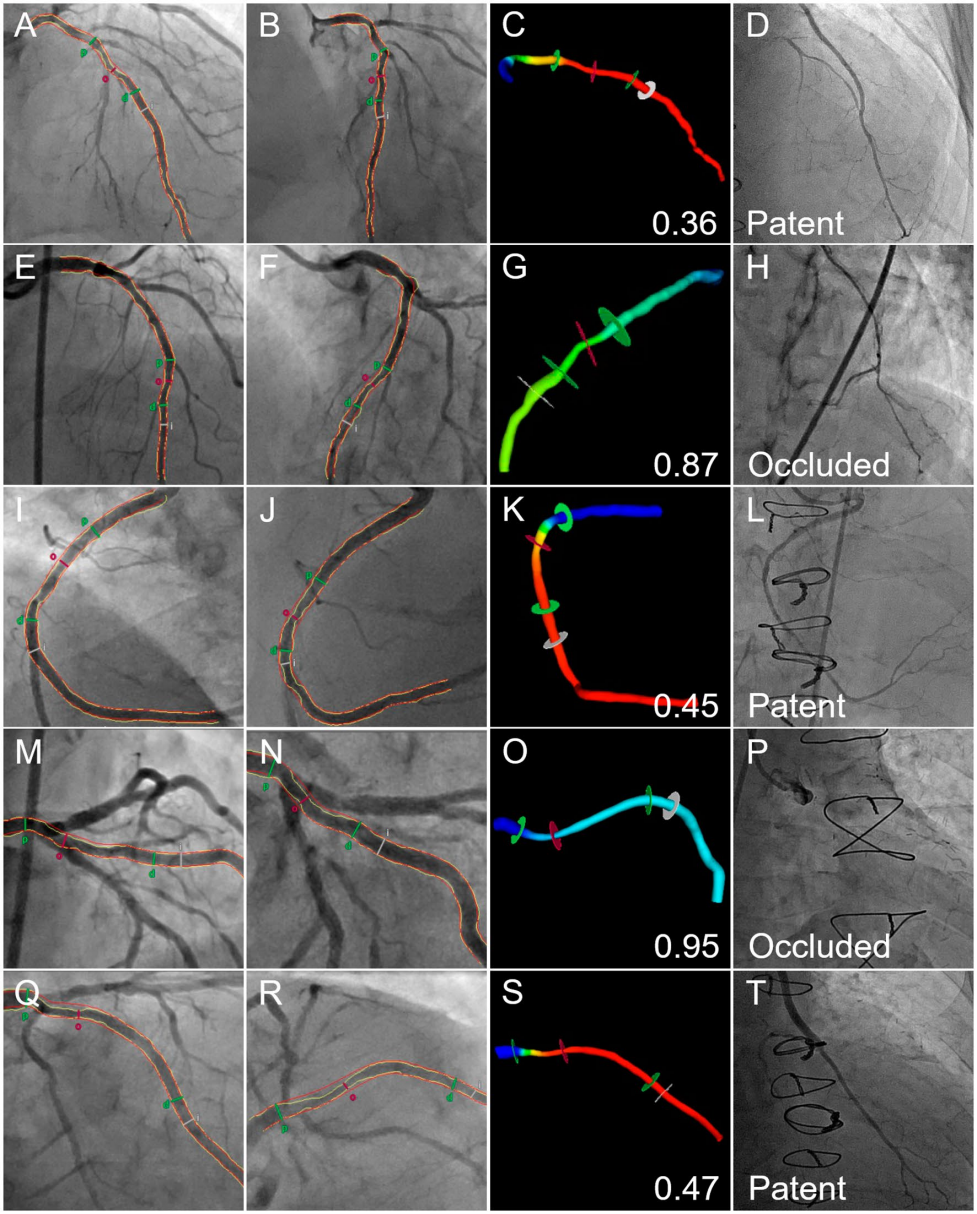
QFR Analysis and Graft Patency. QFR analysis is performed on two angiographic acquisitions (first and second columns), vessel QFR (third column) is recorded and compared with follow-up CTCA or invasive coronary angiography (fourth column). **A**–**D** An LAD with a functionally significant QFR (0.36) and LIMA graft patency. **E**–**H** An LAD artery with a functionally non-significant QFR (0.87) and LIMA skip-graft occlusion. **I**–**L** A PDA with a functionally significant QFR (0.45) and radial graft patency. **M**–**P** A ramus intermedius artery with a functionally non-significant QFR (0.95) and radial graft occlusion. **Q**–**T** An OM with a functionally significant QFR (0.47) with venous graft patency. *LAD* denotes left anterior descending artery, *LIMA* left internal mammary artery, *OM* obtuse marginal artery, *QFR* quantitative flow ratio, and *PDA* posterior descending artery

**Fig. 2:**
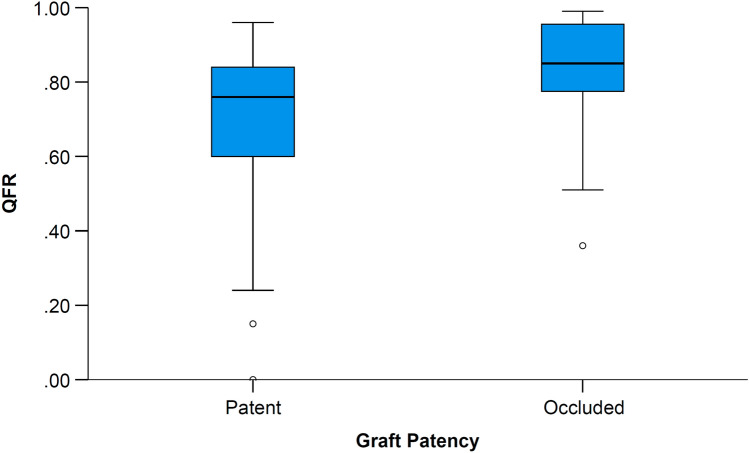
QFR values in patent and occluded grafts. QFR was not statistically significantly higher in occluded grafts

**Fig. 3:**
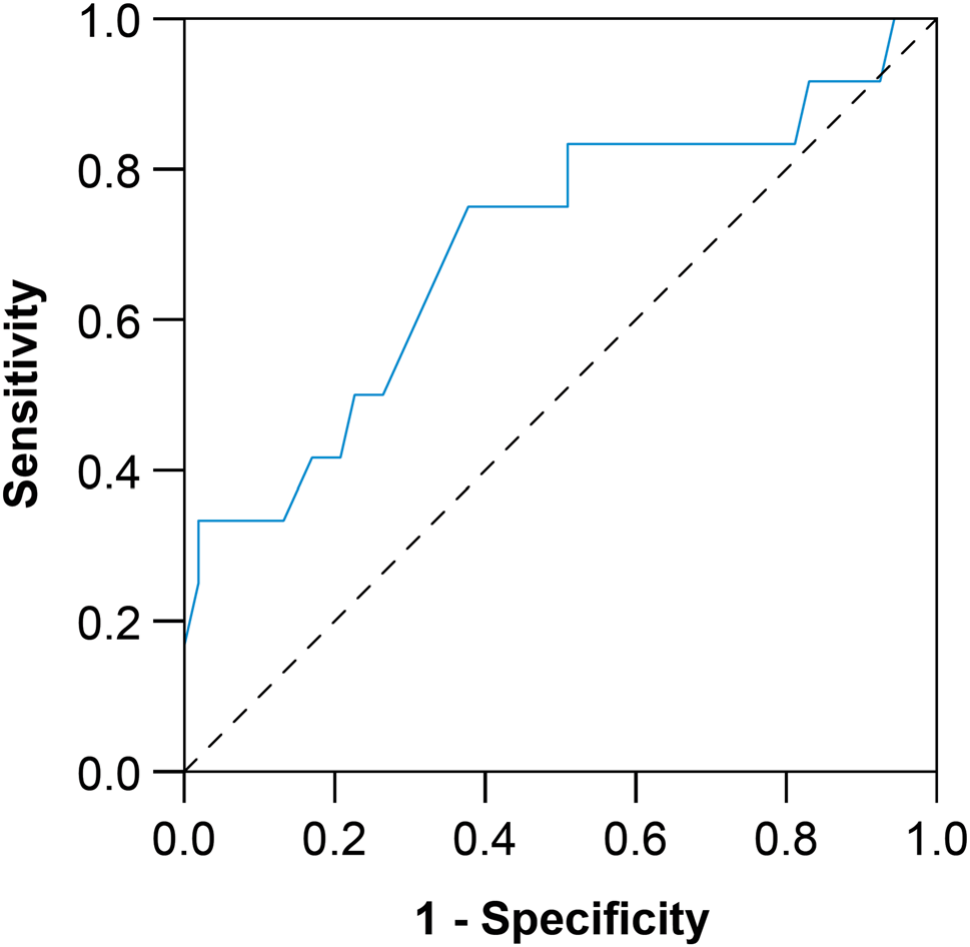
Discriminatory power of QFR to predict graft occlusion. QFR demonstrated a discriminatory power to predict graft occlusion. Diagonal segments are produced by ties

Examples of QFR analysis and graft patency are presented in Fig. 1. QFR was numerically but not statistically significantly higher in occluded grafts (0.81 ± 0.19 vs. 0.69 ± 0.21; P = 0.08) (Fig. 2). QFR demonstrated a discriminatory power to predict graft occlusion (area under the receiver operating characteristic curve [AUC], 0.70; 95% confidence interval [CI] 0.52 to 0.88; P = 0.03) (Fig. 3). The optimal cut-off for predicting graft occlusion was a QFR > 0.80, representing a sensitivity of 75.0%, specificity of 58.5%, positive predictive value of 29.0%, negative predictive value of 91.2% and a diagnostic accuracy of 61.5%. QFR demonstrated a discriminatory power to predict graft occlusion for left-sided (AUC, 0.72; 95% CI 0.53 to 0.92; P = 0.045), but not right-sided vessels (AUC, 0.71; 95% CI 0.33 to 1.00; P = 0.29).

On univariate analysis, a QFR > 0.80 was a predictor for graft occlusion (odds ratio, 4.95; 95% CI, 1.20 to 20.47; P = 0.03) (Table 2).

**Table 2:** Predictors of graft occlusion

Variable	Univariate analysis	
	Odds ratio (95% CI)	P value
QFR > 0.80	4.95 (1.20 to 20.47)	0.03
IVUS minimum lumen diameter > 6.0 mm^2^	0.83 (0.22 to 3.10)	0.83
IVUS diameter stenosis < 2.0 mm	1.68 (0.40 to 4.96)	0.48
IVUS percentage stenosis < 50%	0.76 (0.22 to 2.67)	0.67
QCA lesion length < 10 mm	1.13 (0.26 to 4.94)	0.88
QCA diameter stenosis < 50%	2.07 (0.50 to 8.61)	0.32
QCA minimum lumen diameter > 1 mm	3.45 (0.68 to 17.45)	0.14

*IVUS* denotes intravascular ultrasound, and *QCA* quantitative coronary angiography

**Fig. 4:**
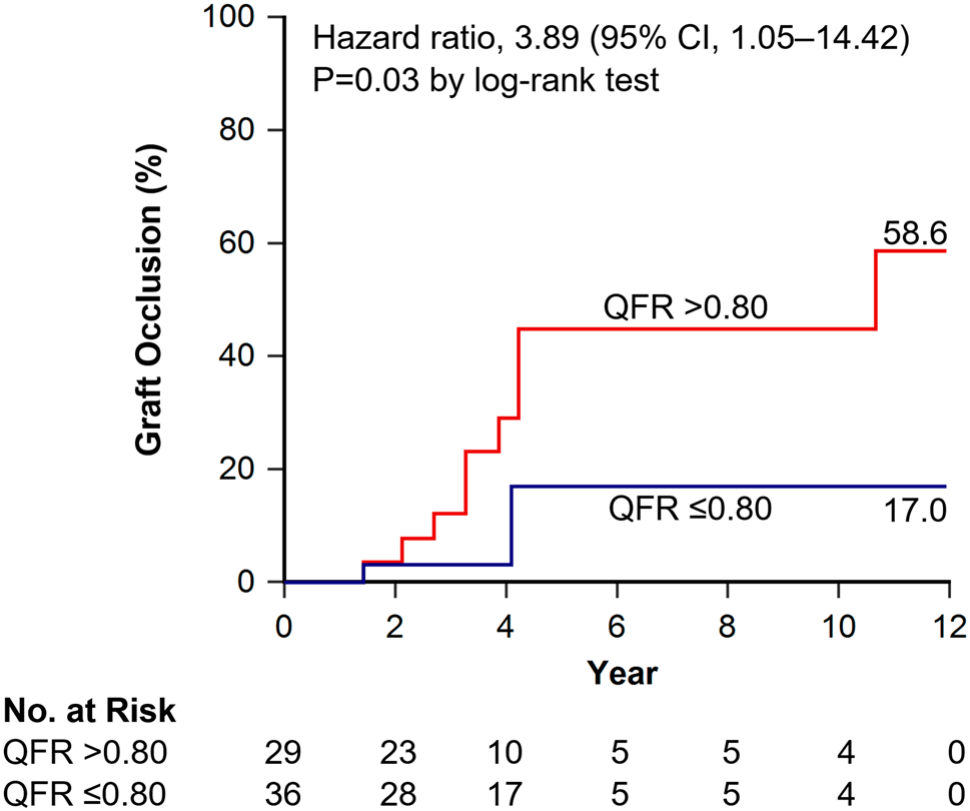
Long-term graft occlusion. Long-term graft occlusion was higher in vessels with a QFR > 0.80, when compared to vessels with a QFR ≤ 0.80

At 5 years, the risk of graft occlusion was higher in vessels with a QFR > 0.80, when compared to vessels with a QFR ≤ 0.80 (44.8% vs.17.0%; hazard ratio, 3.54; 95% CI 0.94 to 13.36; P = 0.04 by log-rank test). At long-term follow-up the risk of graft occlusion was higher in vessels with a QFR > 0.80, when compared to vessels with a QFR ≤ 0.80 (58.6% vs. 17.0%; hazard ratio, 3.89; 95% CI 1.05 to 14.42; P = 0.03 by log-rank test) (Fig. 4).

IVUS MLA (AUC, 0.62; 95% CI 0.47 to 0.76; P = 0.30), diameter stenosis (AUC, 0.52; 95% CI 0.34 to 0.70; P = 0.90) and percentage stenosis (AUC, 0.68; 95% CI 0.41 to 0.95; P = 0.29) did not demonstrate discriminatory power to predict left-sided graft occlusion. There was no correlation between QFR and IVUS MLA (Pearson’s r, 0.03; 95% CI − 0.24 to 0.29; P = 0.84), diameter stenosis (Pearson’s r, 0.06; 95% CI − 0.23 to 0.34; P = 0.68) or percentage stenosis (Pearson’s r, 0.06; 95% CI − 0.42 to 0.51; P = 0.82).

QCA lesion length (AUC, 0.53; 95% CI 0.34 to 0.72; P = 0.76), diameter stenosis (AUC, 0.61; 95% CI 0.43 to 0.78; P = 0.26) and minimum lumen diameter (AUC, 0.65; 95% CI 0.50 to 0.81; P = 0.10) were not predictive of graft occlusion. There was correlation between QFR and QCA lesion length (Pearson’s r, − 0.46; 95% CI − 0.65 to − 0.20; P = 0.001) and diameter stenosis (Pearson’s r, − 0.49, 95% CI − 0.67 to − 0.24; P < 0.001), but not minimum lumen diameter (Pearson’s r, 0.01; 95% CI, − 0.27 to 0.28; P = 0.97).

## Discussion

In this study we investigated whether QFR would predict long-term CABG patency. We found that QFR demonstrated a discriminatory power to predict long-term CABG patency, albeit with somewhat modest diagnostic performance. Furthermore, we showed that whilst QFR was predictive of long-term graft patency, both IVUS assessment of the LMCA and QCA parameters were not predictive of this clinical endpoint.

The usage of physiology to guide percutaneous coronary revascularisation improves clinical outcomes [7–9]. However, the role of physiology in guiding surgical revascularisation is less clear. Both short [2, 10] and long-term [1] CABG patency has been demonstrated in observational studies to be associated with the physiological significance of coronary stenoses, however these results have not been replicated when angiographic and FFR-guided CABG strategies have been assessed in a randomised manner [11, 12]. Reasons for this discrepancy between observation and randomised data might be related to a reluctancy for surgeons to withhold graft placement in angiographically severe but functionally non-significant vessels [12]. Nonetheless, meta-analysis suggests that FFR-guided CABG is associated with a lower rate of graft occlusion [13] and the results of this study support this observation.

FFR-guided CABG has been demonstrated in observation studies to be associated with improved long-term clinical endpoints, including the composite endpoint of death or myocardial infarction [1]. However, when assessed in a randomised manner, angiographically and FFR-guided CABG have similar rates of short [11, 12] and long-term [14] clinical outcomes. Thus, the role of FFR-guided CABG in improving clinical outcomes remains uncertain and further studies will need to evaluate whether QFR-guided CABG might improve patient outcomes.

One potential benefit of FFR-guided CABG is a reduction in the number of bypass grafts placed [11, 12, 15]. This may potentially simplify operating strategy, although it should be noted that aortic cross-clamp time is not reduced using this strategy [11]. In this study a large proportion of vessels (44.6%) did not have a physiologically significant QFR. These results suggest that QFR-guided CABG might reduce the number of bypass grafts placed.

In this study, we performed QFR in patients with visually estimated, angiographically intermediate-grade LMCA disease. The management of this clinical condition is challenging. Invasive physiological functional assessment may be difficult, due to the requirement for catheter disengagement. Furthermore, assessment of FFR in a non-stenosed vessel may be problematic in the presence of co-existing severe proximal disease in the other vessel [16]. Nonetheless, observational data suggest that FFR-guided revascularisation of LMCA disease is associated with favourable long-term clinical outcomes [17]. IVUS may provide further anatomical information and its usage to guide revascularisation has been associated with favourable clinical outcomes [18, 19]. IVUS may also be used to predict functionally significant LMCA disease [20]. However, in this study, there was no correlation between QFR and IVUS parameters.

CABG is associated with a long-term survival benefit for patients with three-vessel coronary artery disease, when compared to angiographically-guided percutaneous coronary intervention (PCI) [21]. However, physiologically-guided PCI may improve outcomes for patients with three-vessel coronary artery disease treated with PCI [22]. Furthermore, QFR may provide refined prognostic risk estimation for patients with three-vessel coronary artery disease [23]. However, the role of physiological assessment in three-vessel coronary artery disease has recently been questioned, as FFR-guided PCI was not found to be noninferior to CABG in regards to the incidence of major adverse cardiac or cerebrovascular events [24]. Therefore, the role of physiological assessment in three-vessel coronary artery disease remains uncertain.

Long-term graft patency differs significantly between radial artery and saphenous venous conduits [25]. In this study, we did not demonstrate any statistically significant difference in the incidence of graft patency between internal mammary, radial artery and saphenous venous conduits. However, given the small number of patients included in this study, the possibility of a type II statistical error cannot be excluded.

It is important to consider how a technology such as QFR might be integrated into the clinical management of patients being considered for CABG. The diagnostic performance of QFR is impaired as QFR values approach the 0.80 cut-off [26]. We would recommend that for patients with QFR in the borderline zone of 0.75 to 0.85, that invasive assessment, potentially with a hybrid strategy of non-hyperaemic indices and FFR could be utilised. This approach could potentially avoid invasive, wire-based assessment in the majority of patients.

It is important to recognise that QFR is just one of several technologies which may be used to predict invasive FFR values. Alternate technologies, such as vessel FFR (vFFR), have recently been shown to demonstrate suitable diagnostic performance for the prediction on invasive FFR measurements [27]. Furthermore, vFFR has been demonstrated to also be predictive of long-term graft patency [28].

Moving forward, the role of QFR-guided revascularisation of patients will need to be assessed prospectively and recently the technology has been demonstrated to improve clinical outcomes in patients with stable angina when compared to angiographically-guided PCI [29]. Furthermore, the FAVOR4-QVAS (NCT03977129) will be assessing whether QFR-guided surgical revascularisation improves clinical outcomes in patients undergoing elective valvular surgery.

### Limitations

It is important to recognise the significant limitations of this study. This was a small single-centre study, on a limited number of patients with only a small number of graft occlusion. The diagnostic performance of QFR to predict long-term graft patency was only modest (diagnostic accuracy 61.5%), potentially limiting the clinical utility of this technology when used in isolation. Invasive FFR measurements were not undertaken, and our study would have been strengthened through the addition of this information, which would have allowed the diagnostic performance of FFR and QFR to be directly compared in this setting. Graft patency was mostly assessed non-invasively and our study would have been strengthened through routine invasive assessment [30]. Our study included two patients with ostial LMCA disease and it should be recognised that QFR has not been validated in this patient cohort. Furthermore, the majority of patients in this study had distal LMCA disease and QFR analysis is no intended for patients with bifurcation lesions with Medina 1,1,1 classification. In this study, QFR was predictive for graft occlusion in left-sided disease, but not in right-sided disease, potentially limiting the utility of this technology, however, confidence intervals were broad, and these discrepant results may potentially represent a type II statistical error. Competitive flow is just one of a number of potential mechanisms for early and late graft failure and these alternate mechanisms were not assessed in this study [31]. Clinical endpoint information was not assessed in this study and the addition of this information would have supported our findings.

## Conclusion

QFR, but not QCA or IVUS parameters, may predict long-term graft patency in patients with LMCA disease undergoing CABG.
